# Redox-dependent modulation of metformin contributes to enhanced sensitivity of esophageal squamous cell carcinoma to cisplatin

**DOI:** 10.18632/oncotarget.18907

**Published:** 2017-07-01

**Authors:** Pin Dong Li, Zhao Liu, Tian Tian Cheng, Wen Guang Luo, Jing Yao, Jing Chen, Zhen Wei Zou, Li Li Chen, Charlie Ma, Xiao Fang Dai

**Affiliations:** ^1^ Cancer Center, Union Hospital, Tongji Medical College, Huazhong University of Science and Technology, Wuhan 430022, China; ^2^ Department of Radiation Oncology, Fox Chase Cancer Center, American Oncologic Hospital, Philadelphia, PA 19111, USA; ^3^ Affiliated Cancer Hospital & Institute of Guangzhou Medical University, Guangzhou 510095, China; ^4^ Department of Radiation Oncology, Anhui Provincial Hospital, Hefei 230001, China

**Keywords:** esophageal squamous cell carcinoma, metformin, cisplatin, chemoresistance, redox

## Abstract

Glutathione is the major intracellular anti-oxidant against reactive oxygen species and serves as a detoxification essential. The anti-diabetic drug metformin has been showed to exert anti-tumor activity via modulation of redox homeostasis. In this study, we provided evidence that metformin inhibits proliferation and induces apoptosis of esophageal squamous cancer cells. Importantly, we found that metformin acts as pro-oxidant via depletion of intracellular glutathione. Co-treatment with metformin reversed the elevated intracellular glutathione induced by cisplatin and therefore enhanced the sensitivity to cisplatin *in vitro* and *in vivo*. Taken together, our data indicate that combination of metformin with cisplatin may represent a novel therapeutic strategy for esophageal squamous cell carcinoma treatment.

## INTRODUCTION

A number of mechanisms underlying cisplatin resistance have been reported. These include resistance to apoptosis, hyperactive DNA repair system or increased detoxification compound [1, 2]. Glutathione (the reduced form: GSH and the oxidized form: GSSG) is the major intracellular anti-oxidant against reactive oxygen species (ROS) and serves as a detoxification essential. Previous reports have demonstrated that glutathione may form adducts with cisplatin [3, 4] and intracellular level of glutathione was associated with cytotoxic effects of cisplatin [5, 6]. It was thus reasonable to hypothesis that agents depleting glutathione may enhance sensitivity of cancer cells to cisplatin.

The anti-diabetic drug metformin is a first-line treatment for type 2 diabetes mellitus patients, which improves insulin resistance and the metabolic syndrome [7–9], two known carcinogenic factors. Recent studies have shown that metformin reduced the risk of developing gastroenterological cancer, including esophageal squamous cell carcinoma (ESCC) in some diabetic patients [9]. It has been well-established that metformin inhibited a variety of human cancer cells *in vitro* and *in vivo*, including breast cancer, colon cancer and gastric cancer [10, 11]. On the other hand, metformin was reported to induce ROS accumulation in cancer cells [12] or serve as ROS scavenger [13].

Esophageal carcinoma is the third most common malignancy of the digestive tracts and the sixth leading cause of cancer death in the world [14–16]. The major histological type of esophageal carcinoma is ESCC, especially in developing countries [15]. Moreover, most ESCC cases is at an advanced stage at diagnosis. Even in the early stage of ESCC, 20% of patients experience a recurrence after curative esophagectomy, which continues to pose a formidable challenge [14, 16, 17]. Platinum-based chemotherapy such as cisplatin is a widely used treatment in a large spectrum of malignancies including ESCC [18]. A majority of cancer patients eventually relapse and develop drug resistance despite initial response to cisplatin [19]. Therefore, novel strategies to enhance sensitivity of ESCC to cisplatin are highly valuable. However, effects of metformin on the redox homeostasis in ESCC cells and on the sensitivity of ESCC cells to cisplatin remain unsolved.

To this end, we explored the redox status in ESCC cells after metformin treatment and found that metformin acts as a pro-oxidant in ESCC. Further analysis indicated that merformin significantly decreased the cell viability, colony formation and induced mitochondria-dependent apoptosis in ESCC *in vitro*. Metformin significantly decreased the intracellular glutathione and enhanced sensitivity of ESCC cells to cisplatin *in vitro* and *in vivo*. Our results revealed that combination of metformin with cisplatin may represent a novel therapeutic strategy for ESCC treatment.

## RESULTS

### Metformin selectively inhibits ESCC cells growth

**Figure 1:**
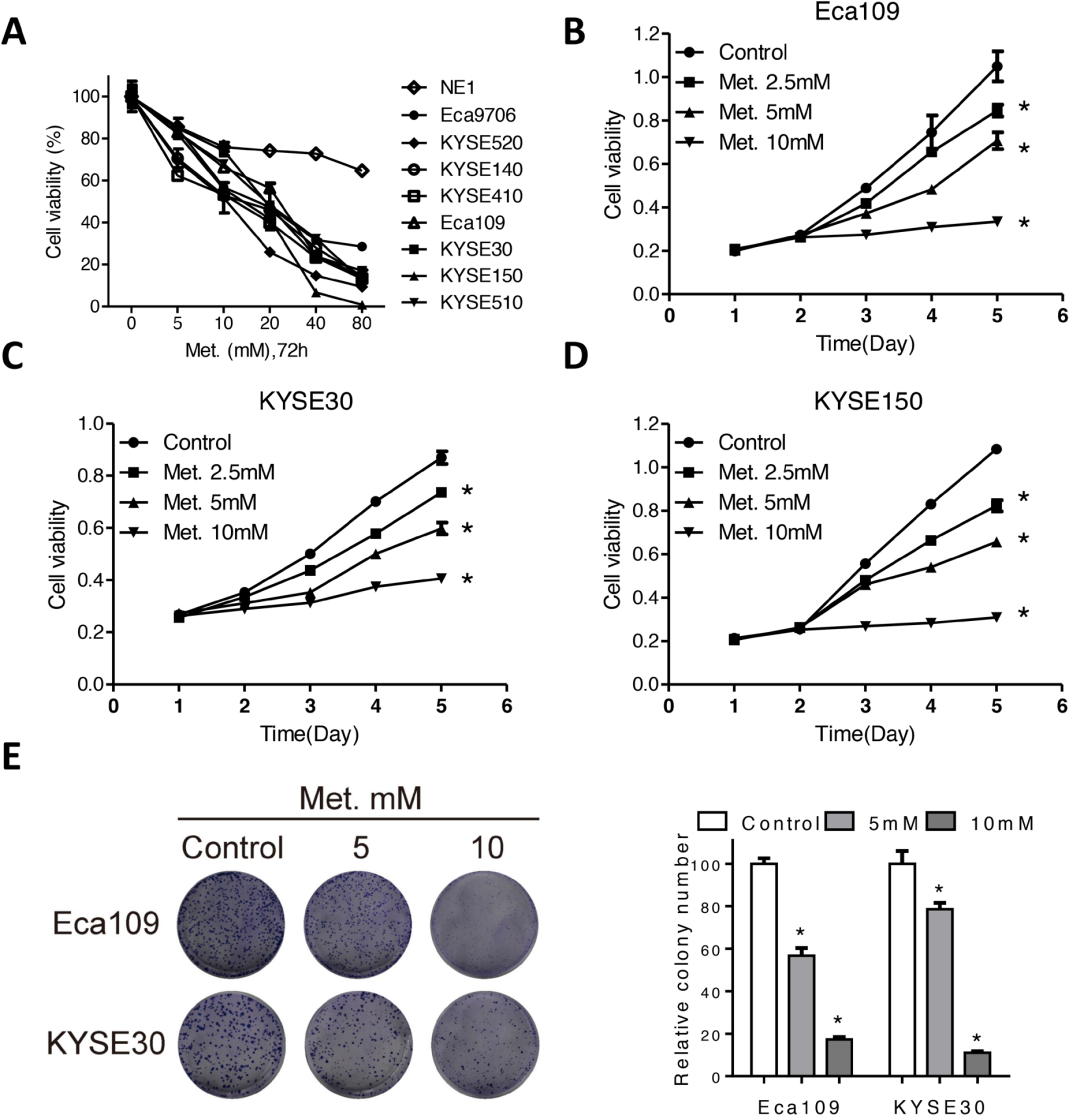
Metformin inhibits cell viability of ECSS cells **(A)** Cell viability of a panel of ESCC cell and one immortalized, noncancerous NE1 esophageal epithelial cell line was detected by CCK-8 kit after treatment with various concentrations of metformin for 72h. Cell viability of Eca109 **(B)**, KYSE30 **(C)** and KYSE150 **(D)** cells treated with metformin at 2.5mM, 5mM and 10mM at indicated time points was detected by CCK-8 kit. **(E)** Representative images (left panel) and quantification (right panel) of colony formation of the Eca109 and KYSE30 cells cultured with metformin at different concentrations for 14 days. Data in **(A, B, C, D** and **E)** are presented as mean ± S.E. (n=3). *P < 0.05 versus corresponding control. Error bars, S.E.

To explore effects of metformin on ESCC, we used a panel of human ESCC cancer cells as well as the immortalized, noncancerous NE1 esophageal epithelial cell line. After treatment with metformin in the dose range of 0-80mM for 72h, the viability of cancer cells significantly reduced to less than 20% of control (Figure 1A), while the viability of NE1 cells was 70% of control cells even after treated with 80mM for 72h (Figure 1A). Moreover, the KYSE30, KYSE150 and Eca109 cells were treated with metformin in 2.5mM, 5mM, 10mM and the viability was detected at different time points. Figure 1B, 1C and 1D showed that metformin decreased cell viability in Eca109, KYSE30 and KYSE150 cells in a time-dependent manner. Cell cycle analysis showed that metformin induced a significant increase of cells in G0/G1 phase (Supplementary Figure 1A) as well as decreased expression of CyclinD1 and elevated expression of p21 (Supplementary Figure 1B) in Eca109 and KYSE30 cells. Consistently, metformin at 5mM significantly decreased the colony formation in Eca109, KYSE30, KYSE150 and KYSE510 cells, while 10mM metformin even completely eliminated colonies of Eca109cells (Figure 1E and Supplementary Figure 1C). On the other hand, metformin exert no inhibitory effects on colony formation of NE1 cells (Supplementary Figure 1D). To sum, our results indicated that metformin selectively kills ESCC cancer cells without any detrimental effects to the noncancerous NE1 cells.

### Metformin induces mitochondria-dependent apoptosis in ESCC cells

**Figure 2:**
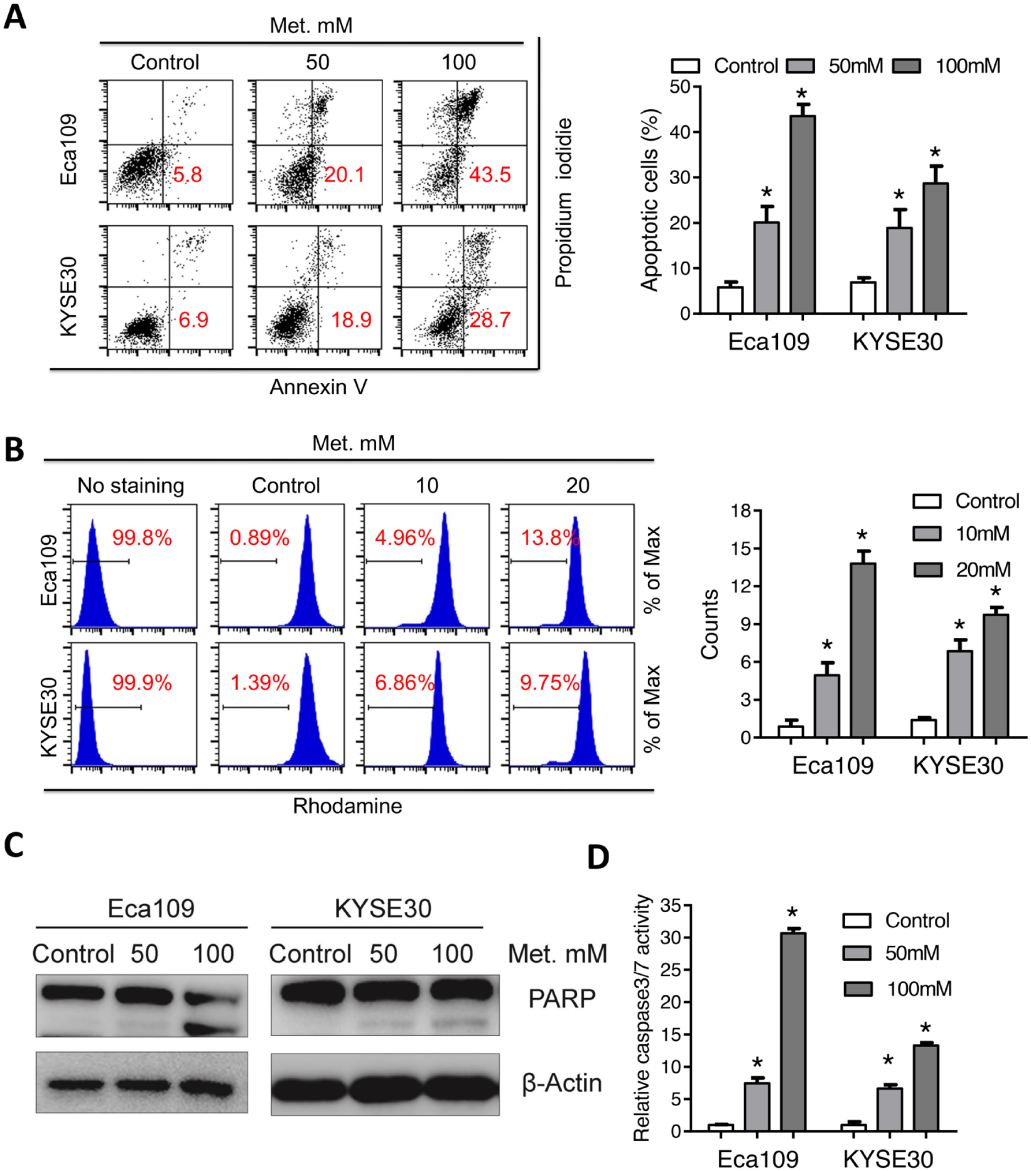
Metformin induces mitochondria-dependent apoptosisof Eca109 and KYSE30 cells **(A)** Eca109 and KYSE30 cells treated with metformin (Control, 50mM, 100mM) for 24h were subjected to the Annexin-V/PI assays. Representative images (left panel) and quantification (right panel) of apoptotic percentages were shown. **(B)** Eca109 and KYSE30 cells treated with metformin (Control, 10mM, 20mM) for 24h were subjected to the rhodamine assays. Representative images of mitochondrial transmembrane potential (left panel) and quantification (right panel) of cells negative for rhodamine staining were shown. **(C)** Immunoblotting of PARP in the indicated cells treated with metformin. β-Actin was used as a loading control. **(D)** Relative caspase 3/7 activity of Eca109 and KYSE30 cells was detected with the Caspase 3/7 Glo assays. Data in A, B and D are presented as mean ± S.E. derived from three individual experiments with triplicate wells. **P < 0.05 versus corresponding control. Error bars, S.E.

To determine whether apoptosis is involved in metformin induced cell death in ESCC, flow cytometer analysis with Annexin V-FITC and propidium iodide (PI) dual labeling was utilized. Metformin induced a dose-dependent increase of apoptotic cell percentage (Figure 2A and Supplementary Figure 2A). As collapse of mitochondria membrane potential (MMP) was associated with apoptosis, we further analyzed change of MMP after metformin treatment with rhodamine staining. Consistently, percentage of cells without rhodamine staining significantly increased after metformin treatment (Figure 2B), indicating that fall of MMP was involved in metformin induced apoptosis in ESCC cells. We further investigated alterations of apoptotic pathways in Eca109 and KYSE30 cells following metformin treatment. Cleavage of PARP (Figure 2C and Supplementary Figure 2B), cleaved caspase3, cleaved caspase7 and cleaved caspase9 (Supplementary Figure 2C) was observed in metformin-treated cells. Moreover, metformin significantly increased the enzymic activity of PARP and caspases (Figure 2C, Supplementary Figure 2D and 2E). Altogether, metformin induced mitochondria-dependent apoptosis in ESCC cells.

### Redox modulation is involved in cytotoxicity of metformin and cisplatin

**Figure 3:**
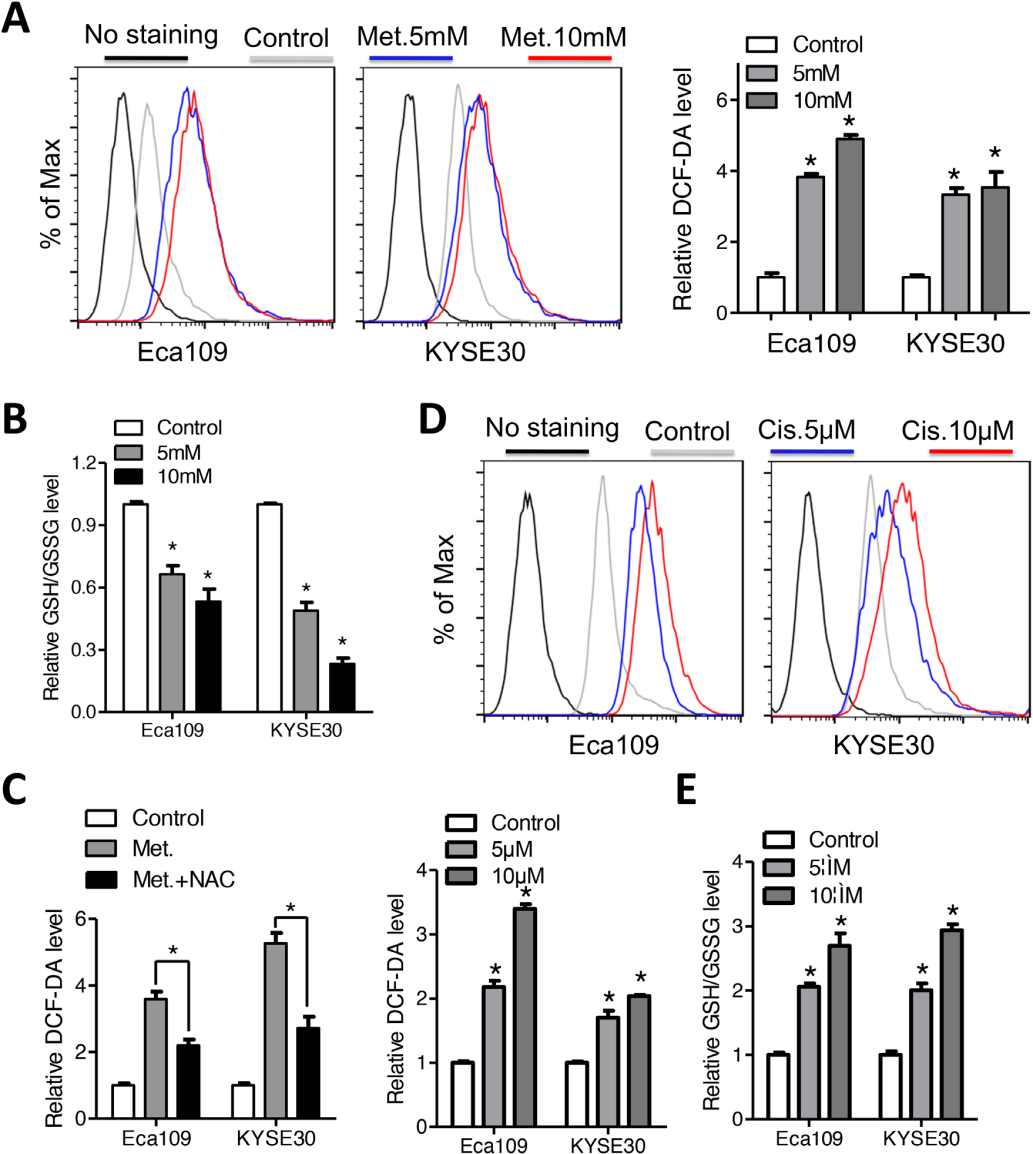
Metformin and cisplatin induces intracellular ROS accumulation in Eca109 and KYSE30 cells **(A)** The intracellular ROS level of Eca109 and KYSE30 cells was monitored by H_2_DCFDA staining after treatment with metformin (Control, 5mM, 10mM) for 12h. The right panel indicated quantification of the fluorescence intensity. **(B)** Eca109 and KYSE30 cells treated with metformin (Control, 5mM, 10mM) for 12h were subjected to GSH/GSSG analysis. **(C)** Eca109 and KYSE30 cells with or without pretreatment with NAC were exposed to metformin (10mM). The intracellular GSH/GSSG level was measured. **(D)** The intracellular ROS level of Eca109 and KYSE30 cells was monitored by H_2_DCFDA staining after treatment with cisplatin (Control, 5μM, 10μM) for 12h. The lower middle panel indicated quantification of the fluorescence intensity. **(E)** Eca109 and KYSE30 cells treated with cisplatin (Control, 5μM, 10μM) for 24h were subjected to GSH/GSSG analysis. Data in **(A, B, C, D** and **E)** are presented as mean ± S.E. derived from three individual experiments with triplicate wells. **P < 0.05 versus corresponding control. Error bars, S.E.

Metformin was reported to act as either anti-oxidant or pro-oxidant in different tumor cells [12, 13]. We therefore analyzed the intracellular redox state after metformin treatment. As shown in Figure 3A, H_2_DCFDA fluorescence intensity in Eca109 and KYSE30 cells was elevated after treatment with metformin for 24h. Consistently, the intracellular glutathione level was reduced by metformin (Figure 3B). However, pretreatment with the NAC, the precursor of glutathione, significantly attenuated the pro-oxidant effects of metformin on ESCC cells (Figure 3C). Expression of NOX1, the major producer of ROS, was elevated after metformin treatment (Supplementary Figure 3A). Previous reports suggested that cisplatin damage DNA via ROS induction and elevated glutathione level significantly decreased cytotoxic efficiency of cisplatin [6]. In accordance with the abovementioned data, we found that the ROS level was significantly increased by cisplatin (Figure 3D). Importantly, the intracellular glutathione level was also elevated after cisplatin treatment (Figure 3E), which may be due to a feedback regulation of ROS induced activation of anti-oxidant system and was further corroborated by a previous report [20]. Together, our data suggest that ROS accumulation was involved, at least in part in the cytotoxic effects of metformin and cisplatin.

### Metformin enhances sensitivity of ESCC cells to cisplatin *in vitro* and *in vivo*

**Figure 4:**
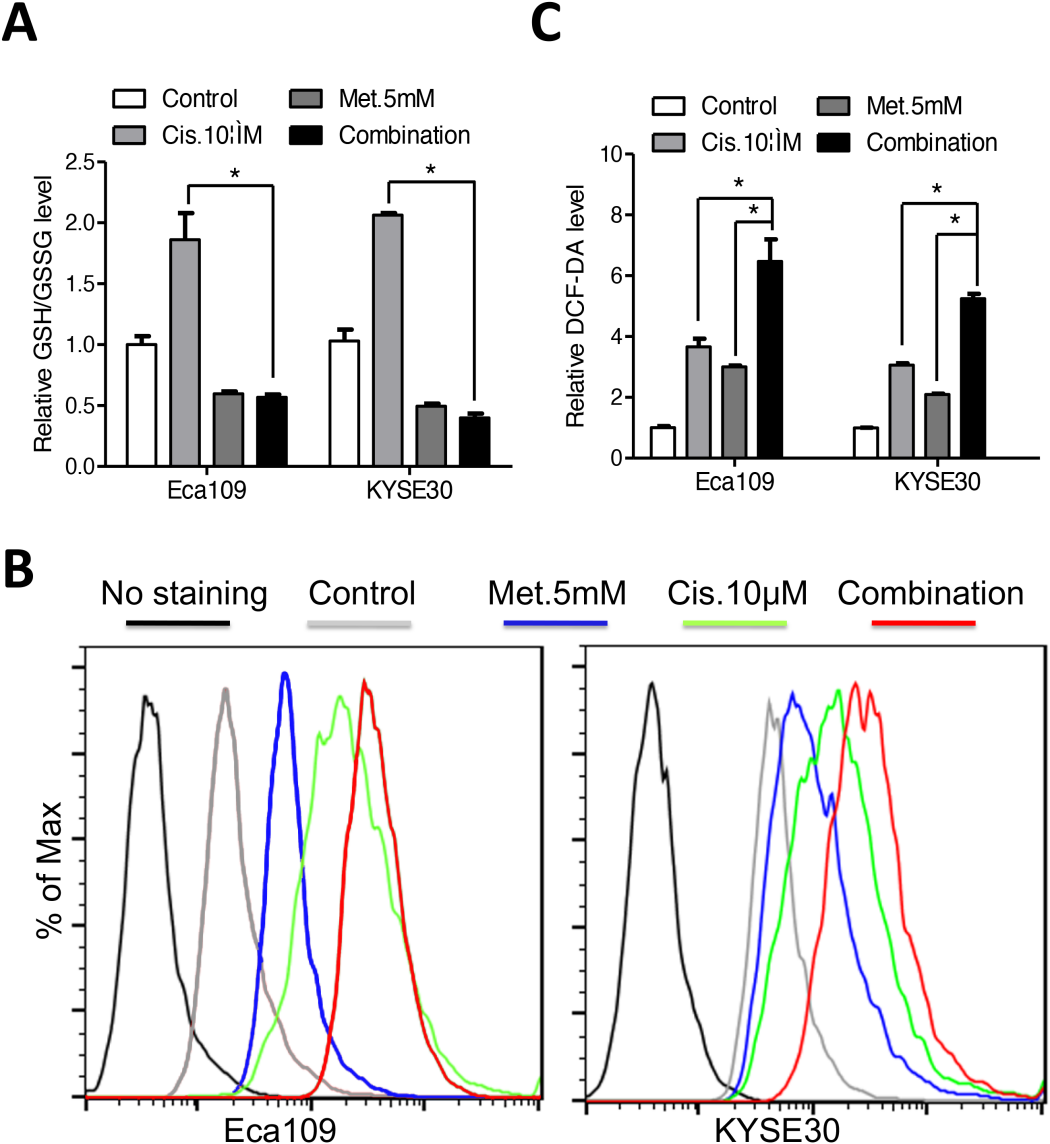
Metformin reversed the elevated glutathione level induced by cisplatin in Eca109 and KYSE30 cells **(A)** Eca109 and KYSE30 cells were treated with metformin (5mM), cisplatin (10μM) or both agents for 12h. The intracellular GSH/GSSG level was measured. **(B)** Eca109 and KYSE30 cells were treated with metformin (5mM), cisplatin (10μM) or both agents for 12h. The intracellular ROS level was monitored by H_2_DCFDA staining. **(C)** Quantification of the fluorescence intensity in Eca109 and KYSE30 cells treated with metformin (5mM), cisplatin (10μM) or both agents for 12h. Data in A and C are presented as mean ± S.E. derived from three individual experiments with triplicate wells. **P < 0.05 versus corresponding control. Error bars, S.E.

**Figure 5:**
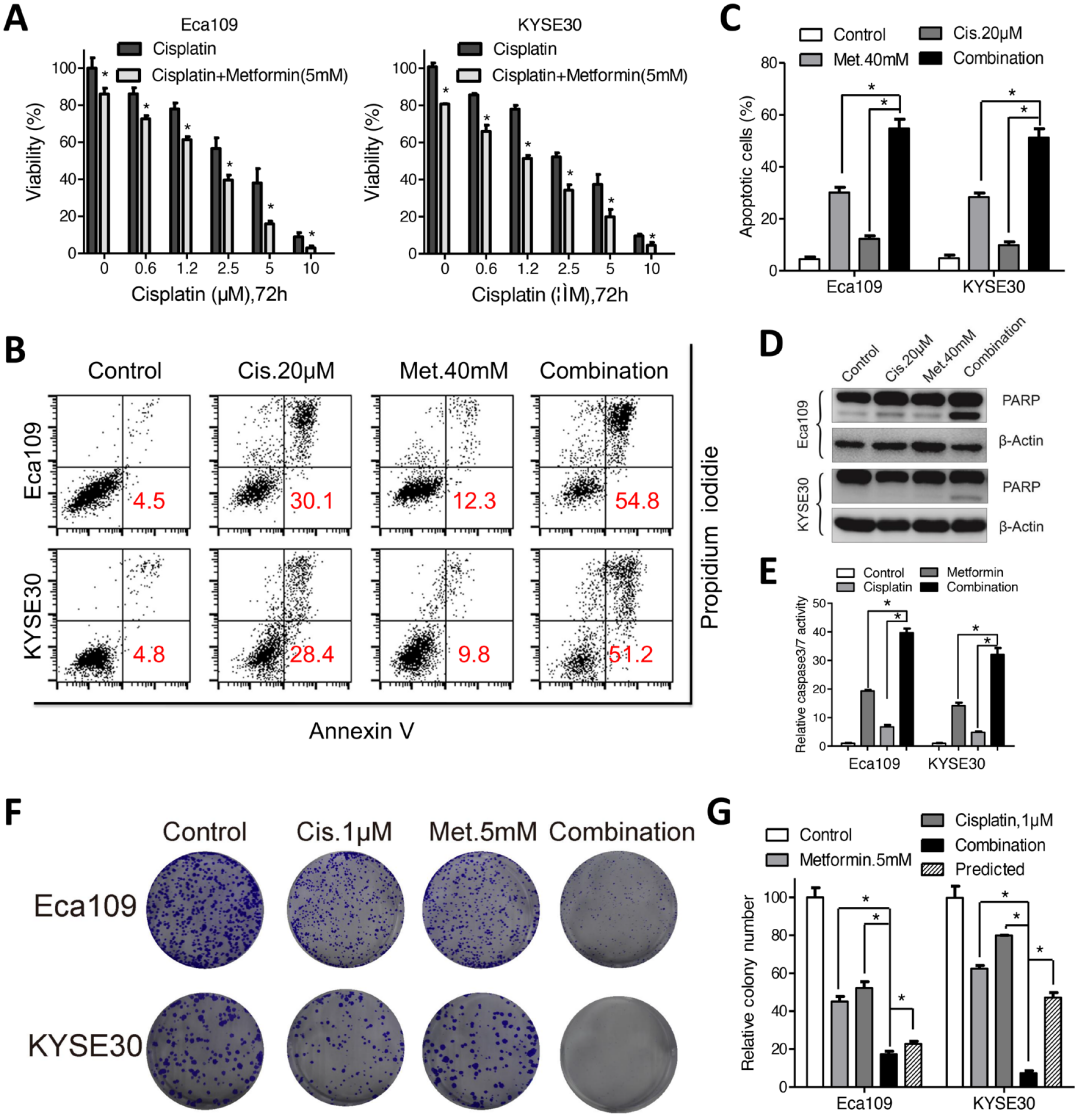
Metformin enhanced sensitivity of Eca109 and KYSE30 cellsto cisplatin **(A)** Eca109 and KYSE30 cells were treated with cisplatin alone or combined with metformin (5mM) at indicated concentrations for 72h. The cell viability was detected by CCK-8 assays. **(B)** Eca109 and KYSE30 cells were treated with metformin (40mM), cisplatin (20μM) or both agents. Cell apoptosis was detected with the Annexin-V/PI assays. Representative images were shown. **(C)** Quantification of dual negative percentage in Eca109 and KYSE30 cells. **(D)** Immunoblotting of PARP in Eca109 and KYSE30 cells treated with the indicated chemicals. β-Actin was used as a loading control. **(E)** Relative caspase 3/7 activity of Eca109 and KYSE30 cells treated with the indicated chemicals was detected with the Caspase 3/7 Glo assays. Representative images **(F)** and quantification **(G)** of colony formation in Eca109 and KYSE30 cells cultured with metformin (5mM), cisplatin (1μM) or both agents for 14 days. The predicted value was calculated by multiplying the relative colony numbers in the cisplatin-treated and metformin-treated samples. The combination effect is considered additive when the observed value is equal to the predicted value. When observed value is less than the predicted value, the combination effect is considered assynergistic. Data in **(A, C, E** and **G)** are presented as mean ± S.E. derived from three individual experiments with triplicate wells. **P < 0.05 versus corresponding control. Error bars, S.E.

Modulation of intracellular glutathione level has been reported to impact the cytotoxic efficiency of platinum compounds [6, 21]. We found that the elevated glutathione level induced by cisplatin was reversed by co-treatment with metformin (Figure 4A). On the other hand, combination of both agents triggered a more dramatic ROS accumulation than either agent alone (Figure 4B and 4C). Expression of NOX1 was synergistically upregulated by combination of metformin and cisplatin (Supplementary Figure 3B). We therefore hypothesis that metformin may enhance sensitivity of ESCC cells to cisplatin via redox modulation. First, combination of metformin with cisplatin significantly decreased the cell viability of ESCC cells than cisplatin alone (Figure 5A). Combination index calculated with the Calcusyn software was less than 1, indicating synergistic effects between metformin and cisplatin (Supplementary Figure 4A). Apoptosis analysis demonstrated that apoptotic cells was significantly increased in the combination group than either agent alone (Figure 5B, 5C and Supplementary Figure 4B). Immunoblotting showed remarkable elevated cleavage of PARP (Figure 5D and Supplementary Figure 4C) and caspases (Supplementary Figure 4D) when treated with metformin and cisplatin. Consistently, combinational treatment in Eca109 and KYSE30 cells significantly stimulated enzymic activity of PARP (Supplementary Figure 4E) and caspases (Figure 5E and Supplementary Figure 4F). MMP was significantly reduced by co-treatment of metformin and cisplatin (Supplementary Figure 4G and 4H). Metformin plus cisplatin significantly decreased the colony formation in Eca109 and KYSE30 cells (Figure 5E and 5F).

**Figure 6:**
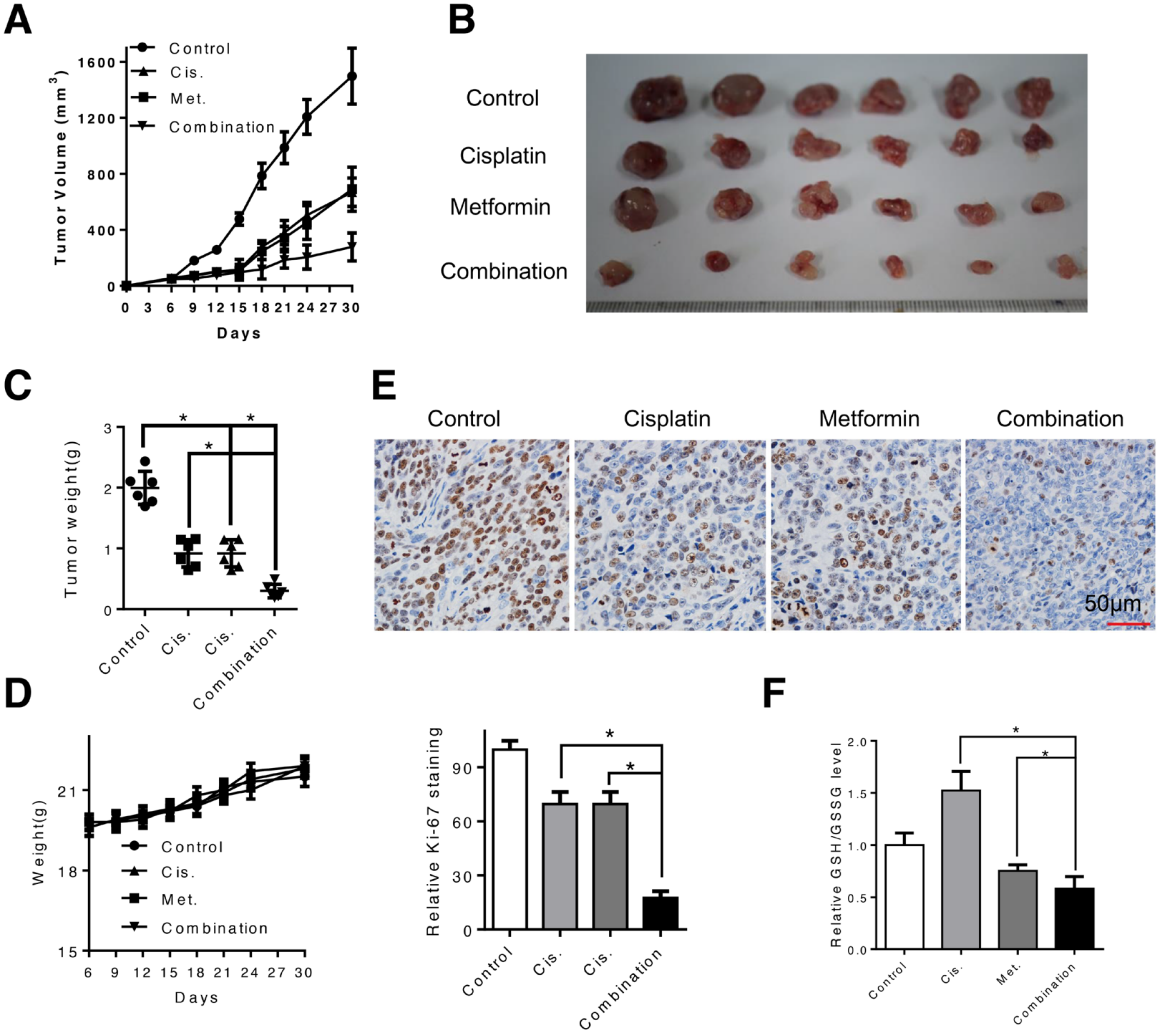
Combination of metformin and cisplatin inhibited ESCC tumor growth *in vivo* **(A** and **B)** Tumor growth curve of control (PBS), metformin-treated (250mg/kg, once per day), cisplatin-treated (4mg/kg, once per week) and both agents-treated mice. **(C)** Weight of dissected tumor was measured. **(D)** Weight of mice during the treatment period was recorded. **(E)** Representative images of immunohistochemical analysis of Ki-67 in dissected tumors. Scale bars: 100μm. **(F)** The dissected xenografts were subjected to GSH/GSSG analysis. Data in **(A, C, D, E** and **F)** are presented as mean ± S.E. **P < 0.05 versus corresponding control. Error bars, S.E.

We proceed to evaluate the effects of metformin, alone or in combination with cisplatin *in vivo* in a subcutaneous xenograft tumor model. When the tumors volume reached 50mm^3^ after inoculation of Eca109 cells, mice were randomly assigned to four groups and given daily i.p. injections of metformin (250mg/kg), cisplatin (4mg/kg, once per week) or both agents. Consistent with the *in vitro* results that metformin decreased the growth of ESCC cells and synergized with cisplatin to induce cytotoxicity, administration of metformin significantly suppressed the tumor growth in nude mice and combination of metformin and cisplatin resulted in more effective inhibition of tumor growth than either agent alone (Figure 6A and 6B). The tumor weight was significantly decreased by combined treatment (Figure 6C). Moreover, the mice did not show any visible side effects or changes in body weight during the course of treatment (Figure 6D). Immunohistochemistry analysis with an antibody against Ki-67, a marker of cell proliferation, showed that Ki-67-positive cells were significantly decreased in the combination group compared with that in the metformin- or cisplatin-treated group (Figure 6E). Elevated glutathione level in the cisplatin treated xenografted samples was reversed by co-treatment with metformin (Figure 6F), which is consistent with the *in vitro* results. Taken together, our data suggest that metformin enhance sensitivity of ESCC cells to cisplatin *in vitro* and *in vivo*.

## DISCUSSION

Mountains of studises have explored the anti-neoplastic effects of metformin in human cancers including ESCC, although the underlying mechanisms remain elusive [12, 22–24]. Interestingly, metformin may act as pro-oxidant [25] or anti-oxidant [13, 23] in cancer cells. Our study demonstrated that metformin induced ROS accumulation in ESCC cells and depleted the major anti-oxidant glutathione. Cheng G et al. reported that metformin decreased the intracellular ROS via NOX4 in pancreatic cancer cells [13]. Inhibition of STAT3 and NF-κB/HIF-1α signaling was also associated with anti-proliferation activities of metformin [26, 27]. These results indicate the multi-faceted roles in the cancer therapy.

Platinum-based chemotherapy such as cisplatin has been widely used in the clinic for a broad spectrum of tumors including ESCC [28]. However, acquired resistance often leads to therapy failure and disease relapse. Increased glutathione level has been associated with cisplatin resistance through forming the DNA-Platinum adducts or scavenging the toxic oxygen species induced by cisplatin [3, 21]. Our data provided evidence that metformin at relative high concentration induced accumulation of ROS and thus depletion of intracellular glutathione resulting in enhanced sensitivity of ESCC cells to cisplatin treatment. Synergistic effects of metformin and cisplatin have been observed in gastric cancer [29], lung cancer [30] as well as oral oral squamous cell carcinoma [26], although the precise mechanisms were tumor specific. Interestingly, other researchers have drawn seemly total different conclusions from ours. Damelin LH et al. showed that reducing state in ESCC cells induced by metformin protects esophageal SCC cells (WHCO1, WHCO5, SNO) from cisplatin treatment [23]. This discrepancy was understandable as metformin activates glutathione production in WHCO1, WHCO5, SNO cells [23] while accelerates glutathione consumption in Eca109 and KYSE30 cells leading to apposite sensitivity to cisplatin. Pharmaceutical effect as well as chemosensitizing roles of metformin was dependent on culture conditions [30–32] and cell types [24, 26, 27, 32, 33]. Yu Hongliang et al. found that metformin induced enhanced cytotoxicity of cisplatin only in glucose-deprivation medium [31]. We found that metformin at relatively high concentration synergizes with cisplatin via reversion of elevated intracellular glutathione induced by cisplatin. Combination of metformin and cisplatin induced significantly elevated ROS level than either agent alone. As it has been reported that glucose deprivation leads to elevation of intracellular ROS level [34], it is thus reasonable to conclude that metformin showed combinational effects with cisplatin in ESCC under circumstances that induce ROS stress. Moreover, in the nude mice model, we and others [24, 35] observed metformin significantly suppressed tumor development and enhanced sensitivity to cisplatin treatment.

The key findings of our present study provide comprehensive insights into the therapeutic application of metformin via induction of glutathione depletion mediated ROS accumulation in ESCC *in vitro* and *in vivo*. Moreover, metformin enhanced sensitivity of ESCC cells to cisplatin. Overall, our data suggest that combination of metformin with cisplatin may represent a novel therapeutic strategy for ESCC treatment.

## MATERIALS AND METHODS

### Reagents and antibodies

Metformin and cisplatin were purchased from Selleck (Huston, USA). 2',7'-Dichlorofluorescein diacetate (H_2_DCFDA) and N-Acety-L-Cysteine(NAC) was purchased from Sigma (St. Louis, MO, USA). Antibodies against CyclinD1, p21, PARP, caspase3, caspase7, caspase9 and β-Actin were purchased from Cell Signaling Technology (Beverly, MA, USA). The anti-NOX1 antibody was purchased from Abcam (Cambridge, Massachusetts, USA). The Dulbecco’s modified Eagle’s medium (DMEM), Roswell Park Memorial Institute (RPMI1640) and fetal bovine serum were obtained from Gibco (Life Technologies, New York, USA). Crystal violet staining and rhodamin were obtained from Beyotime Institute of Biotechnology (Shanghai, China).

### Cell lines and culture conditions

Human ESCC cell lines KYSE520, KYSE140, KYSE410, KYSE30, KYSE150 and KYSE510 were obtained from Deutsche Sammlung von Mikroorganismen und Zellkulturen (DSMZ), the German Resource Centre for Biological Material. The ESCC cell lines Eca109, Eca9706 and esophageal epithelial cell NE1 was a kind gift from Dc. Guan XY from Sun Yat-sen University Cancer Center. The cells were grown in Dulbecco’s modified Eagle medium (Invitrogen, Carlsbad, California, USA) supplemented with 10% fetal bovine serum (HyClone, Logan, Utah, USA), 100 unit/ml penicillin and 100 unit/ml streptomycin at 37°C in a humidified atmosphere with 5% CO_2_. Immortalized NE1 cells were cultured in Defined Keratinocyte-SFM (DK-SFM)/Eplife mixed medium (Life Technologies, Carlsbad, California, USA). All experiments were performed during the exponential phase of cell growth.

### Cell viability and colony formation assay

Cell viability was performed according to a previous report [22]. Briefly, cells in the exponential phase were trypsinized and plated in the 96-well plates at a density of 3000 cells in 100μl medium per well. The cells were treated with metformin at the indicated concentrations 24h later. The cell viability was detected with the CCK-8 kit (Dojindo, Japan) at the indicated time points. For the colony formation assay, cells at a density of 250/ml were plated in the 6-well plates (in triplicate) 48h before metformin was added to the medium. The cells were allowed to colonize for 14 days. To visualize colonies, the culture media were removed and the cells were fixed with methanol for 15min and stained with crystal violet staining solution. The colonies were dried on the air, photographed and colonies with more than 50 cells were counted under the microscopy.

### Apoptosis and cell cycle analysis

Apoptosis was quantified with an Annexin V-FITC/PI apoptosis detection kit (Beyotime Institute of Biotechnology, China) as described by the manufacturer’s instructions. Briefly, after exposure todrugs for 48h at the indicated time points, the cells were collected and washed with PBS, gently resuspended in Annexin V binding buffer and incubated with Annexin V-FITC/PI. Flow cytometry was performed using Cellquest software (BD Biosciences, San Jose, CA, USA). Cell cycle analysis was performed according to a previous report [35]. Mitochondrial membrane potentialchanges were investigated using the rhodamine staining. Frequency plots without rhodamine staining were counted to explore the effects of metformin on mitochondrial membrane potential. Also, caspase activity was measured by Caspase 3/7 Glo assay (Promega, Madison, WI, USA) according to the manufacturer’s protocol.

### Assessment of ROS levels and intracellular GSH/GSSG

The ESCC cells were plated on a 6-well plate the day before treatment. Media was then replaced with fresh media containing cisplatin or metformin or both. Following exposure to the drug, ROS levels were assessed by incubating cells with the H_2_DCFDA (10μM; Life Technology) for 30 min at 37°C. The hydrogen peroxide (2μM) was added to the labeled cells 5 minutes before fluorescence measurement to serve as the positive control. Then the cells were washed twice and resuspended in PBS and assessed for fluorescence intensity by employing the flow cytometer. Data were analyzed using Flow Jo software. The intracellular GSH/GSSG level was measured with the GSH/GSSG-Glo™ kit (Promega, Madison, WI, USA) according to the manufacturers’ instructions.

### Western blot analysis

Standard western blotting was done as previously described [35]. Briefly, whole-cell lysates were prepared from cells at the indicated times after treatment with metformin, cisplatin or both. Cell lysates were resolved by SDS/PAGE and transferred electrophoretically to PVDF membrane (Millipore, Billerica, MA, USA). The membranes were probed with specific antibodies and the immunoreactive proteins were detected by the enhanced chemiluminescene (ECL) kit (Santa Cruz, CA, USA).

### Animal study

All the animal experiments were done according to an Institute Animal Care and Use Committee-approved protocol. Twenty-four female nude mice (4-5 weeks old) were purchased from the Guangdong Province Laboratory Animal Center (Guangzhou, China). The Eca109 cells were collected, washed twice with cold PBS, counted and suspended at a final concentration of 2×10^7^/ml. The mice were inoculated subcutaneously in the flanks with 100μl cells. When the tumors were measurable, the experimental group was treated daily with intraperitoneal (i.p.) injections of metformin (250mg/kg), while the control group received equal volume of vehicle only. For cisplatin and co-treatment experiment, the mice were treated with i.p. injections of cisplatin (4mg/kg, once per week), alone or in combination with daily i.p. injections of metformin. The treatment lasted for 4 weeks and the mice weights were monitored every three days. The length and width of the tumors were measured using calipers every three days and the tumor volume was calculated using the formula: length × (width)^2^× 0.5.

### Statistical analysis

All statistical analyses were performed using the SPSS17.0 statistical software package (SPSS Inc., Chicago, IL, USA). Comparisons between two groups were performed using Student’s t-test. As for comparisons among more than two groups, one-way ANOVA and Newman Keul’s multiple comparison tests were used. Data represent the Mean ± S.E. The Calcusyn Biosoft (Ferguson, MO, USA) was used to calculate combination index of metformin with cisplatin. The *P*-value of 0.05 was considered statistically significant.

## SUPPLEMENTARY MATERIALS FIGURES


