# Supplementary material for: Redox-dependent modulation of metformin contributes to enhanced sensitivity of esophageal squamous cell carcinoma to cisplatin
Source: Oncotarget. 2017 Jul 1;8(37):62057–68. doi: 10.18632/oncotarget.18907 (PMC5617486; doi:10.18632/oncotarget.18907)
Supplement: Supplementary file 1 [file oncotarget-08-62057-s001.pdf]

Redox-dependent modulation of metformin contributes to enhanced sensitivity of esophageal squamous cell carcinoma to cisplatin

SUPPLEMENTARY MATERIALS

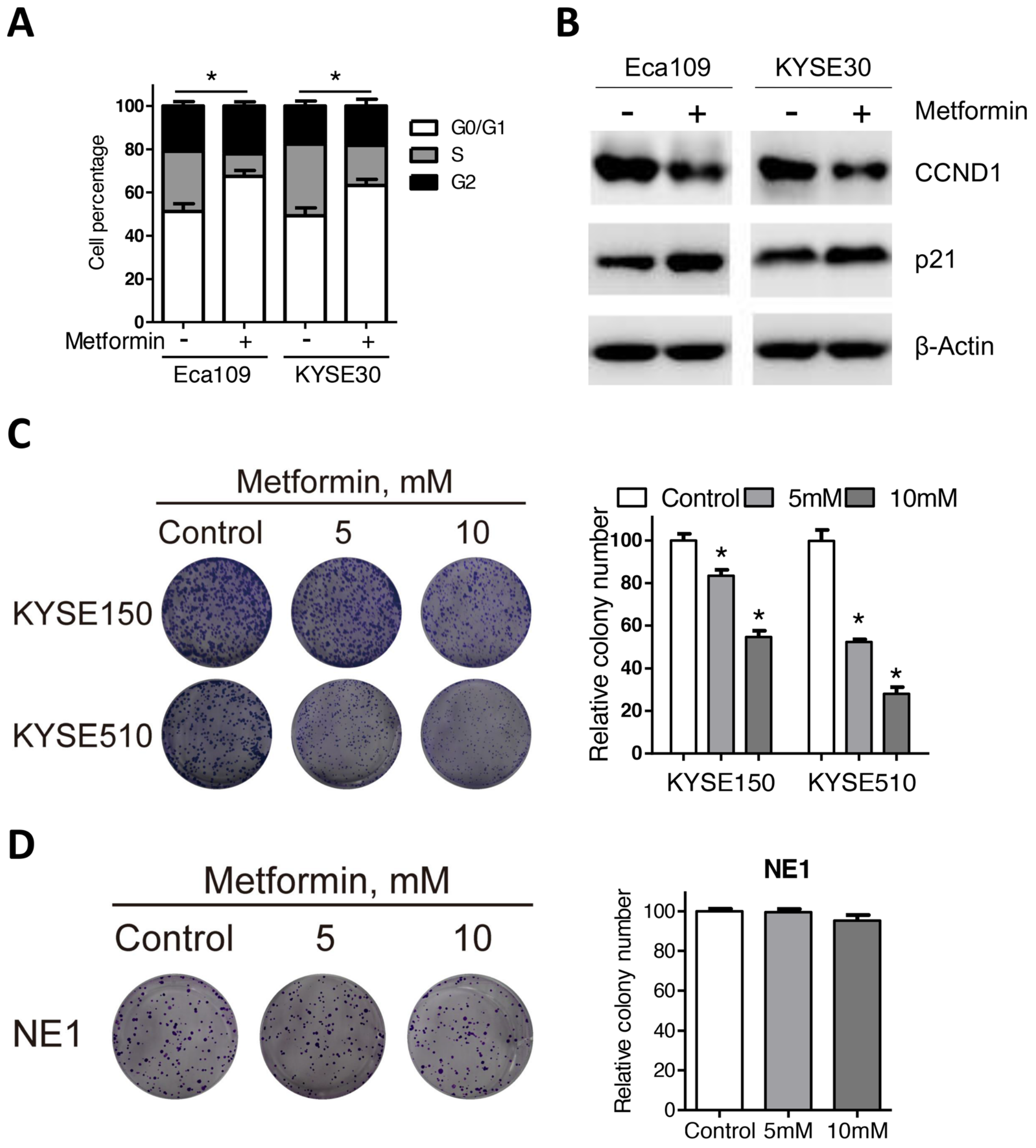

Supplementary Figure 1: Metformin inhibits colony formation and induces G0/G1 arrest of ECSS cells. **(A)** Cell cycle distribution of Eca109 and KYSE30 cells after treatment with metformin (5mM, 24h) was detected with cytometer. **(B)** Expression of CCND1 and p21 in Eca109 and KYSE30 cells after treatment with metformin (5mM, 24h) was detected by western blot. β-Actin was used as loading control. **(C)** Representative images (left panel) and quantification (right panel) of colony formation of the KYSE150 and KYSE510 cells cultured with metformin at different concentrations for 14 days. **(D)** Representative images (left panel) and quantification (right panel) of colony formation of NE1 cells cultured with metformin at different concentrations for 14 days. Data in **(A, C** and **D)** are presented as mean ± S.E. (n=3). *P < 0.05 versus corresponding control. Error bars, S.E.

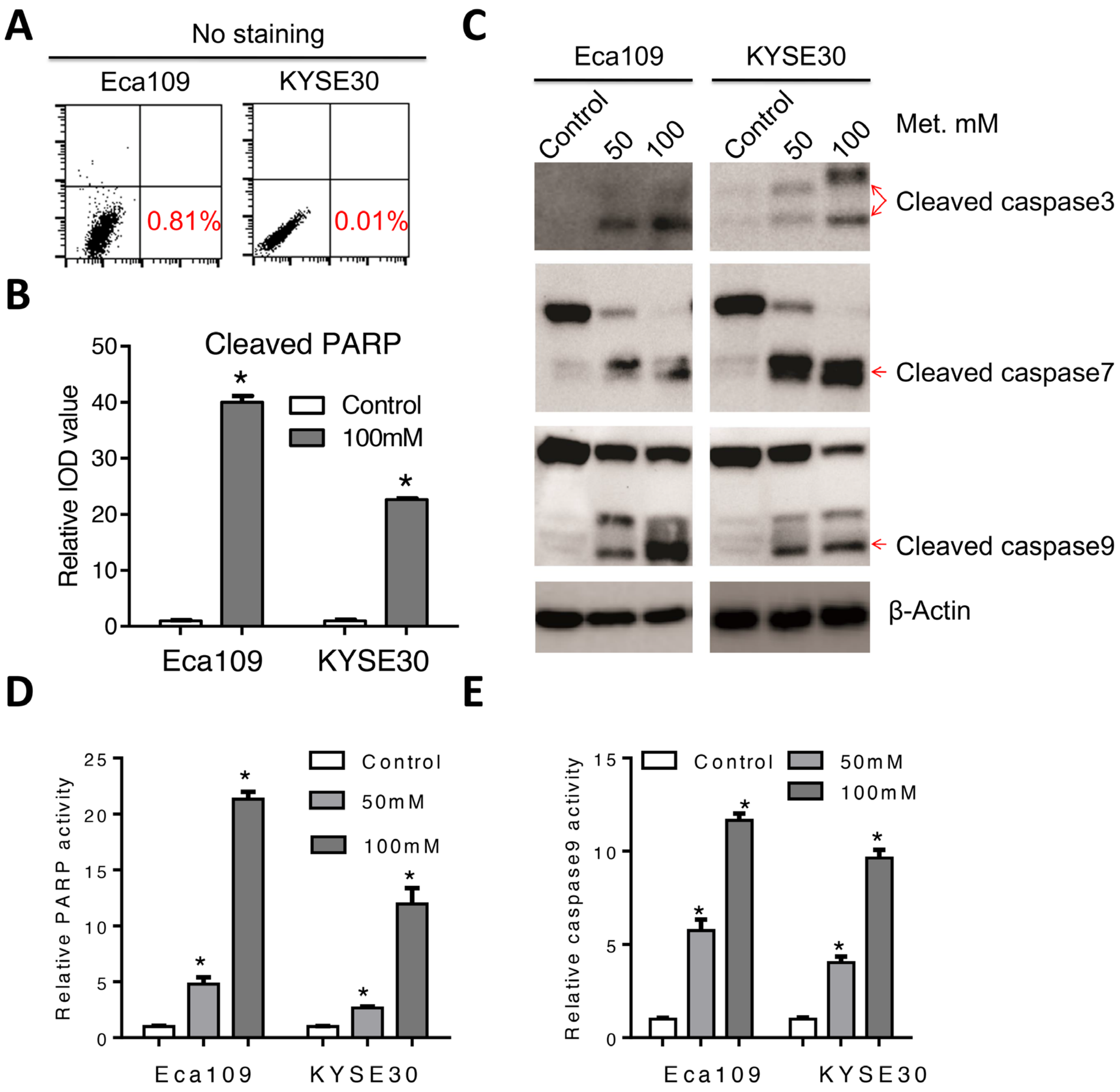

Supplementary Figure 2: Metformin induces apoptosis of ESCC cells. (A) Negative controls for flow analysis in Figure 2A. **(B)** Quantification of cleaved PARP in the indicated cells treated with metformin. **(C)** Immunoblotting of cleaved caspase3, cleaved caspase7 and cleaved caspase9 in the indicated cells treated with metformin. β-Actin was used as a loading control. **(D)** Relative PARP activity of Eca109 and KYSE30 cells was detected with the PARP Glo assays. **(E)** Relative Caspase9 activity of Eca109 and KYSE30 cells was detected with the Caspase9 Glo assays. Data in **(B, D** and **E)** are presented as mean ± S.E. (n=3). *$P < 0.05$ versus corresponding control. Error bars, S.E.

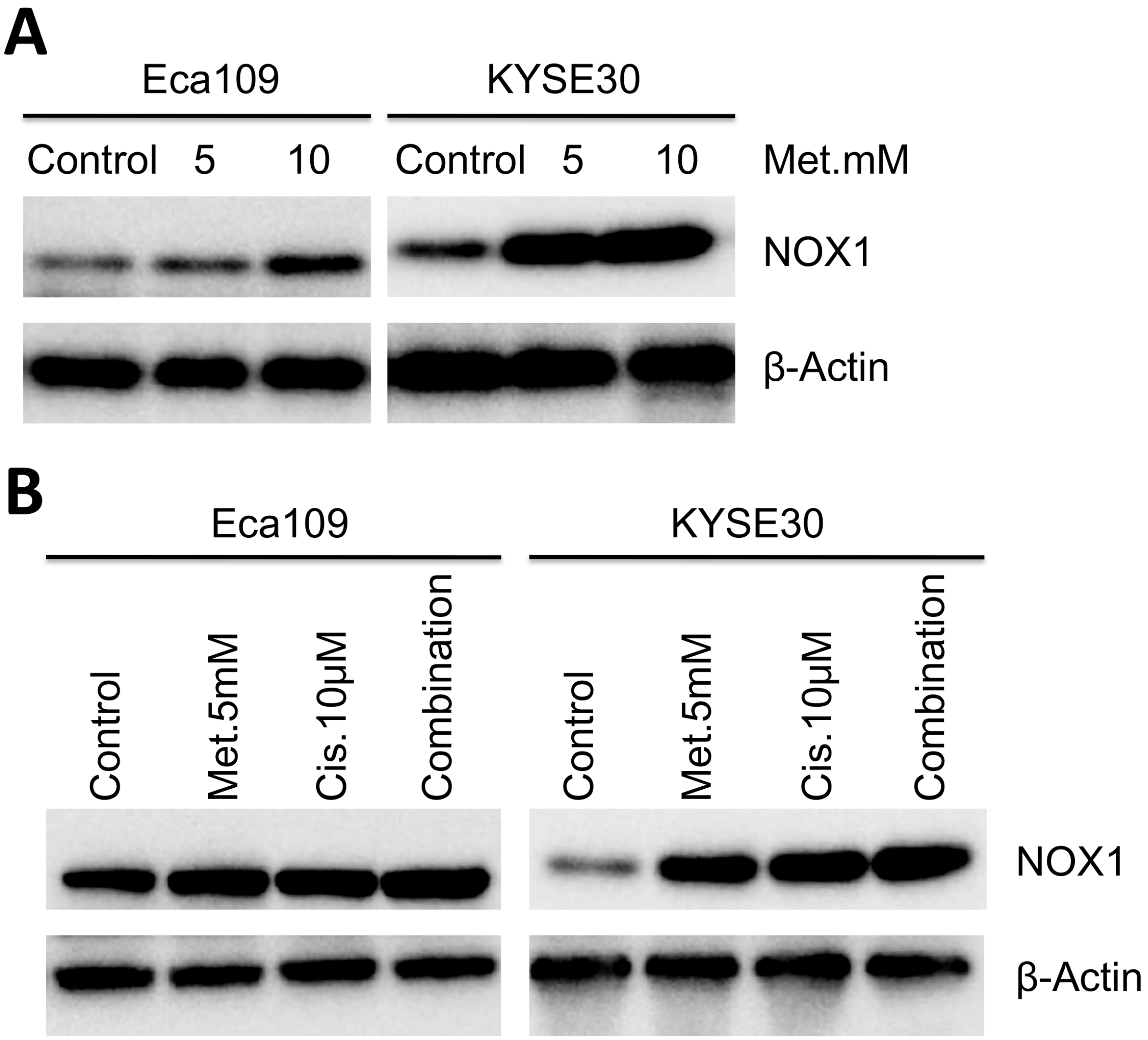

Supplementary Figure 3: Metformin and cisplatin induces expression of NOX1. (A) Immunoblotting of NOX1 in the indicated cells treated with metformin. β-Actin was used as a loading control. **(B)** Immunoblotting of NOX1 in the indicated cells treated with the indicated medicals. β-Actin was used as a loading control.

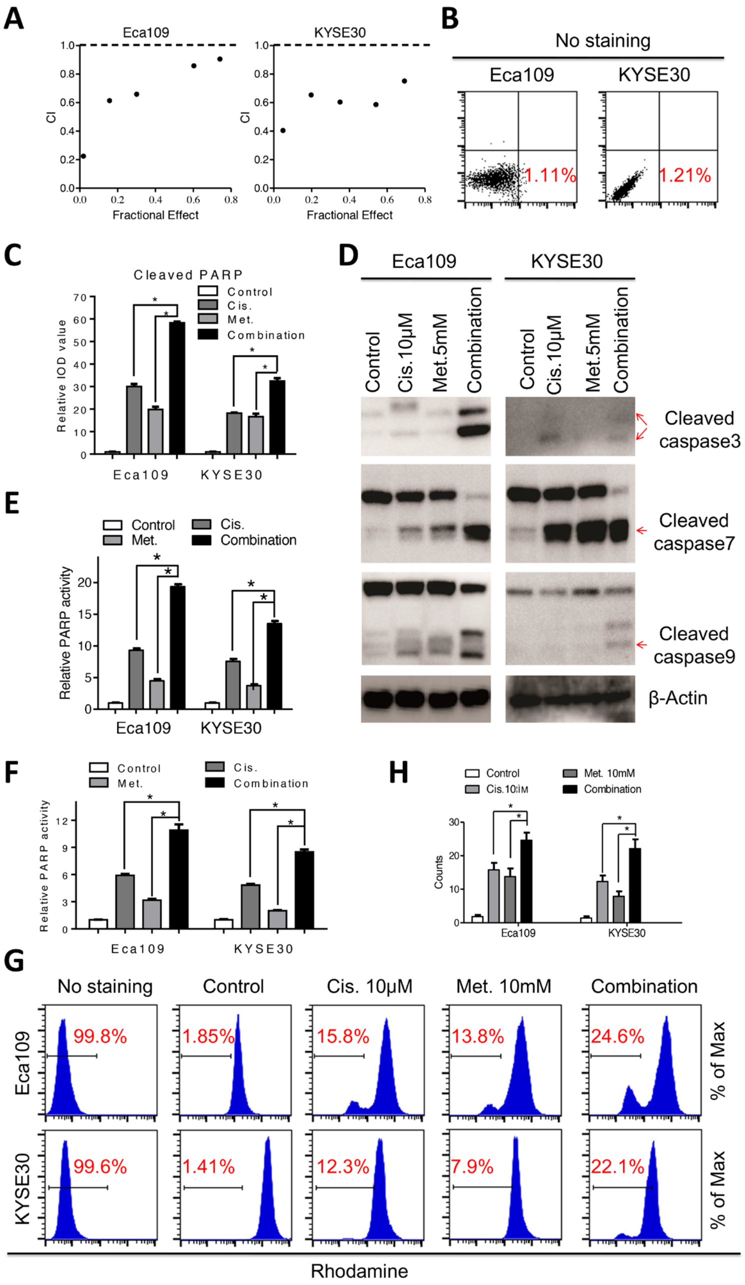

Supplementary Figure 4: Metformin synergize with cisplatin. **(A)** The combination index (CI) of metformin and cisplatin treatment in Eca109 and KYSE30 cells was analyzed using a median dose-effect method with CalcuSyn software (Biosoft). CI = 1 indicates an additive effect, CI < 1 indicates a synergistic effect and CI > 1 indicates an antagonist effect. **(B)** Negative controls for flow analysis in Figure 5B. **(C)** Immunoblotting of cleaved caspase3, cleaved caspase7 and cleaved caspase9 in the indicated cells treated with treated with the indicated medicals. β-Actin was used as a loading control. **(D)** Quantification of cleaved PARP in the indicated cells. **(E)** Relative PARP activity of Eca109 and KYSE30 cells was detected with the PARP Glo assays. **(F)** Relative Caspase9 activity of Eca109 and KYSE30 cells was detected with the Caspase9 Glo assays. Eca109 and KYSE30 cells were treated with metformin (10mM), cisplatin (10μM) or both agents for 24h were subjected to the rhodamine assays. Representative images **(G)** and quantification **(H)** of cells negative for rhodamine staining were shown. Data in **(D, E, F and H)** are presented as mean ± S.E. derived from three individual experiments with triplicate wells. **P < 0.05 versus corresponding control. Error bars, S.E.
